# Uncovering a multitude of human glucocorticoid receptor variants: an expansive survey of a single gene

**DOI:** 10.1186/s12863-019-0718-z

**Published:** 2019-02-08

**Authors:** Stacey M. Leventhal, Debora Lim, Tajia L. Green, Anna E. Cantrell, Kiho Cho, David G. Greenhalgh

**Affiliations:** 1 Shriners Hospitals for Children Northern California, Sacramento, California USA; 2 Department of Surgery, University of California, Davis, Sacramento, California USA

**Keywords:** Glucocorticoid receptor, Polymorphisms, Alternative splicing, Locus specific database, Insertions, Deletions

## Abstract

**Background:**

Glucocorticoids are commonly used in the clinical setting for their potent anti-inflammatory effects; however, significant variations in response to treatment have been demonstrated. Although the underlying mechanisms have yet to be fully understood, this variable response may be a result of alterations in human glucocorticoid receptor (hGR) expression and function. In addition to hGRα, the biologically active isoform, a screening of current databases and publications revealed five alternative splice isoforms and hundreds of variants that have been reported to date. Many of these changes in the hGR-coding gene, NR3C1, have been linked to pathophysiology. However, many studies focus on evaluating hGR expression in vitro or detecting previously reported variants.

**Results:**

In this study, blood from healthy volunteers, burn and asthma patients, as well as from peripheral blood mononuclear cells isolated from leukoreduced donor whole blood, were screened for NR3C1 isoforms. We identified more than 1500 variants, including an additional 21 unique splice isoforms which contain 15 new cryptic exons. A dynamic database, named the Universal hGR (UhGR), was created to annotate and visualize the variants.

**Conclusion:**

This identification of naturally occurring and stress-induced hGR isoforms, as well as the establishment of an hGR-specific database, may reveal new patterns or suggest areas of interest that will lead to the improved understanding of the human stress response system.

**Electronic supplementary material:**

The online version of this article (10.1186/s12863-019-0718-z) contains supplementary material, which is available to authorized users.

## Background

Glucocorticoids are a class of steroid hormones associated with many biological processes including inflammation and immune response. Synthetic glucocorticoids have become one of the most common pharmacologic therapies for conditions such as asthma, autoimmune disorders, and sepsis. However, the clinical use of glucocorticoids has been complicated by hypersensitivity, drug resistance, and pervasive side effects [1–4]. During a post-injury systemic inflammatory response, the glucocorticoid pathway is essential to mounting the anti-inflammatory response needed to return the body to homeostasis [5]; however, there can be wide variations in individual responses to seemingly identical physiologic stressors. These variations may be influenced, in part, by an individual’s regulation and expression of human glucocorticoid receptor (hGR) isoforms. Improved understanding of hGR expression and function may offer solutions to the limitations of therapeutic glucocorticoids and aid in understanding the pathophysiology of disease [6].

hGR is a ligand-inducible and ubiquitously expressed nuclear hormone receptor that is part of the hypothalamic-pituitary-adrenal (HPA) axis, where it is involved in attenuating the stress response. Being lipophilic, glucocorticoids diffuse across the cellular membrane where they bind to hGR, their intracellular receptor that resides primarily unbound in the cytoplasm [7]. The glucocorticoid/hGR complex undergoes conformational changes and translocates to the nucleus where it binds to glucocorticoid response elements (GREs) and then interacts with co-regulators that assist with hGR’s transcriptional activities [7].

**Fig. 1:**
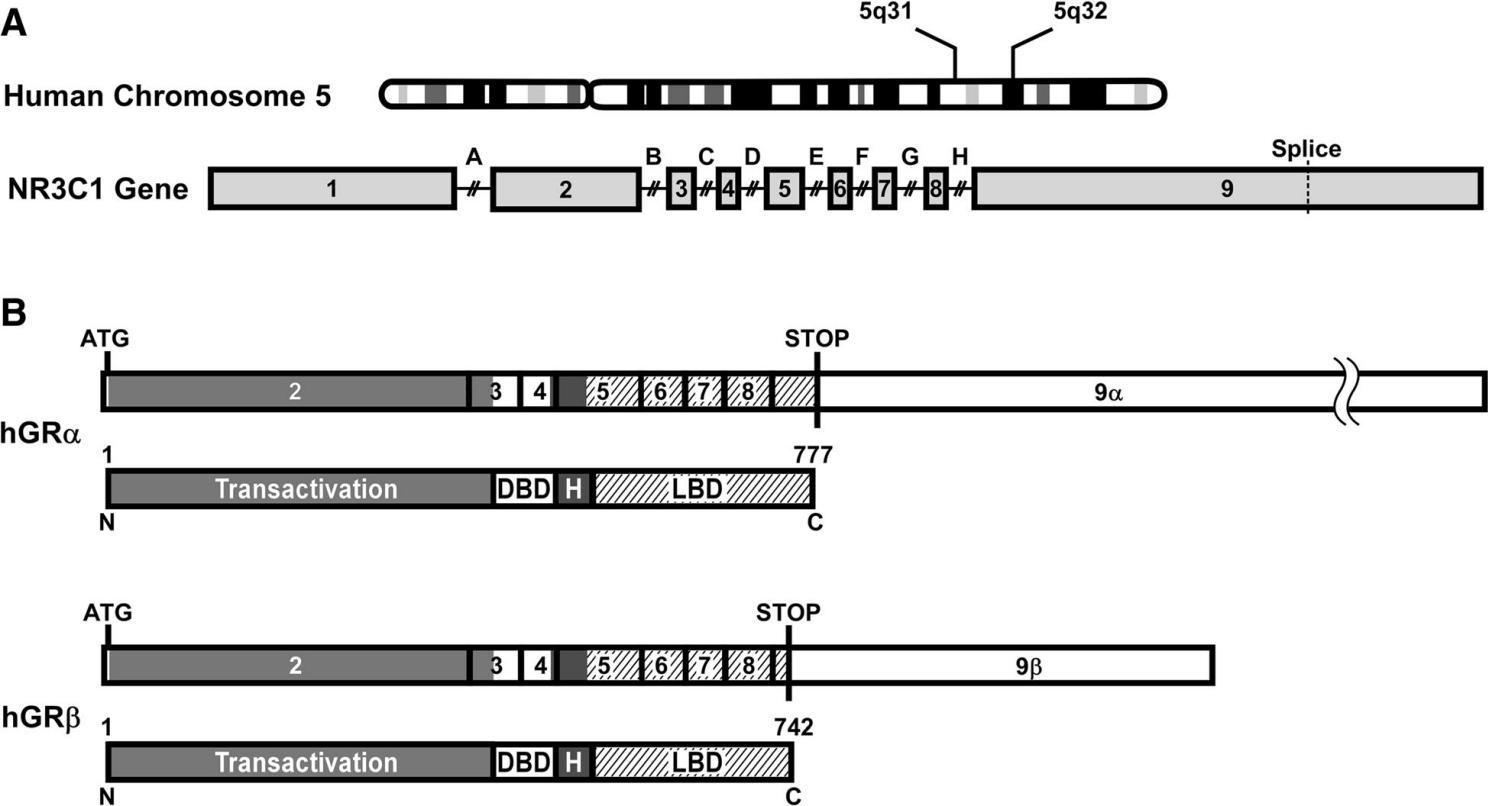
Diagram of NR3C1: the human glucocorticoid receptor (hGR) gene. **a** The hGR gene, NR3C1, is located on Chromosome 5q31–32 and is composed of one untranslated exon (exon 1) and eight coding exons (2–9). Alternative splicing of exon 9 results in two isoforms, hGRα and hGRβ, and the splice junction is indicated with a vertical dashed line. Relative intron (A → H) locations are shown above the gene. **b** hGRα, the biologically active isoform, codes for a 777 amino acid protein that is made up of four domains: transactivation domain, DNA-binding domain (DBD), hinge region (H), and ligand-binding domain (LBD). hGRβ arises from alternative splicing of exon 9 to produce a 742 amino acid protein where the last fifteen amino acids differ from hGRα. Shaded areas in the exons match the different domains. Start and stop codons are indicated on the coding sequence with vertical lines

The hGR is encoded by the NR3C1 gene, located on Chromosome 5 (5q31–32) [8], and consists of nine exons [9], of which most of exons two through nine are translated (Fig. 1a). Due to alternative splicing, there are multiple variants of the non-coding exon one [10], as well as two variants of exon nine. The biologically active isoform is hGRα, which codes for a 777 amino acid protein that is composed of four domains: a transactivation domain, a DNA-binding domain, a hinge region, and a ligand-binding domain (Fig. 1b) [11]. Since 1985, when hGRα and its dominant negative inhibitor (hGRβ) were first identified [9, 12], additional alternative splice isoforms (hGRγ, hGR-A, hGR-P) and hundreds of novel single nucleotide polymorphisms (SNPs) have also been recognized [13–15].

Several alternative splice variants of hGR have been found to have pathologic implications. hGRβ, which differs from hGRα due to the alternative splicing of exon 9, has been linked a multitude of diseases such as glucocorticoid-insensitive asthma [16], pediatric acute lymphoblastic leukemia [17], glucocorticoid-resistant ulcerative colitis [18], and cancer [19–21]. High expression levels of hGRγ, a variant that contains an additional alanine, has been associated with exogenous glucocorticoid resistance in children with acute lymphoblastic leukemia [22]. Another splice variant, hGR-P, terminates in the retained intron G and has been found to be expressed in both normal cells and those derived from subjects with Cushing’s syndrome and several cancer types, including multiple myeloma, acute lymphoblastic leukemia, and non-Hodgkin’s lymphoma [23, 24]. Additionally, late-stage glucocorticoid-resistance cells, derived from an immortalized multiple myeloma line, had greater expression of hGR-P than in cells representing early-stage resistance, indicating the isoform potentially could contribute to resistance development [25]. However, despite numerous studies that have linked alternative splice variants to a variety of diseases, their specific roles have yet to be fully understood.

In addition to splice variants, single nucleotide changes can also have a profound physiological impact. Two of the most commonly studied SNP sites (N363S and ER22/23EK) cause altered, but opposing, responsiveness to glucocorticoids in vivo [26]. The A1088G SNP (rs56149945, NM_000176.2:c.1088A > G), which results in the amino acid change N363S, has been found to increase sensitivity to glucocorticoids [27] which can increase an individual’s risk for medical conditions such as coronary artery disease [28], obesity [29], and other illnesses. Contrastingly, a combination of two SNPs (GAG|AGG → GAA|AAG) produces the E22E and R23K isoform, denoted as ER22/23EK (rs6189/rs6190, NM_000176.2:c.[66G > A;68G > A]), which has been linked to glucocorticoid insensitivity and reportedly confers such health benefits as a decreased risk of dementia [30] and lower insulin and LDL cholesterol levels [31].

Many papers have reported numerous hGR variants but because of the scale and complexity of the gene, it is difficult to track the rapidly expanding list of the identified sequence changes. Although several databases exist to catalog genome-wide variants, studies have shown that curated databases which focus on specific genes (or ‘locus-specific databases’ [LSDBs]) have twice as many unpublished variants as published ones, indicating the need for LSDBs in genetic research [32]. Unfortunately, hGR lacks a comprehensive and up-to-date LSDB. We believe that the extreme diversity already discovered in the NR3C1 gene warrants the need for a modified LSDB system that would allow for both dynamic annotation and analysis of variations. This database would be a valuable tool for identifying correlations between hGR variants and an individual patient’s response to both injury and glucocorticoid treatment.

To better understand the impact of hGR variations, we have explored the NR3C1 gene in two populations: volunteers (i.e., healthy adults) and people suffering from burns or asthma (i.e., medical conditions that have variable responsiveness to glucocorticoid treatment). Several isoforms have already been described but our laboratory has identified many more. These variants, in addition to those already published in journal articles and public databases, serve as the foundation for a new LSDB-style system created for the curation and analysis of data, named the Universal hGR.

## Results

### Alternative splice isoforms

**Fig. 2:**
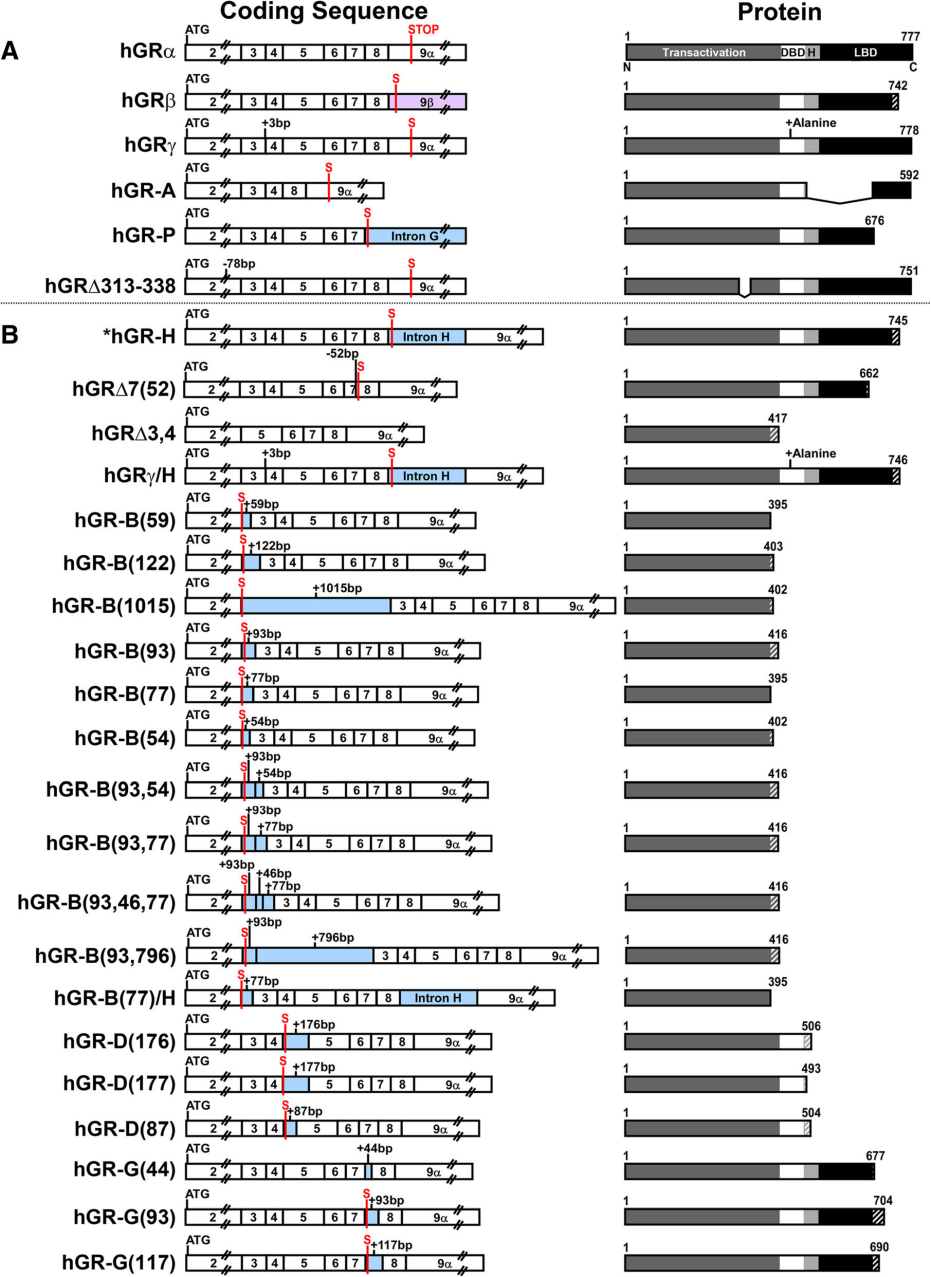
Coding sequences and protein structures of splice variants. **a** The coding sequences and putative protein structures for hGRα and the five published splice variants of hGR are shown. Vertical lines indicate the location of the start codon (ATG) and stop codon (S), along with the four domains: transactivation, DNA-binding domain (DBD), hinge region (H) and ligand-binding domain (LBD). Protein structures have amino acid lengths noted and sequence variations relative to hGRα are indicated with hatched lines. **b** Diagram of the coding sequences and putative protein structures for the 21 splice variants identified by our laboratory. *Previously published as hGR-S1 [35]

To survey for published splice variants, PubMed and several NR3C1 LSDBs were searched, and five alternative splice variants identified (Fig. 2a). hGRβ arises from alternatively spliced exon 9, resulting in a 742 amino acid protein, of which the first 727 match the 777 amino acids in hGRα [12]. First identified in 1999, hGRγ reportedly comprises 3.8–8.7% of normal GR expression and has three base pairs (+GTA) retained between exons 3 and 4, introducing an extra arginine in the DNA-binding domain (DBD) that reduces the transcriptional activation of hGRα by half [14]. hGR-P and hGR-A were both discovered in 1993 in a glucocorticoid-resistant human multiple myeloma cell line [15]. hGR-P retains a portion of intron G, which contains an early stop codon that results in a 676 amino acid protein, of which amino acids 1–674 match hGRα, and is missing a portion of the ligand-binding domain (LBD). hGR-A lacks exons 5 through 7, generating a 592 amino acid protein that has a truncated hinge region and LBD. First reported in 2007, hGR Δ313–338 has a 78 nucleotide deletion in exon 2 that results in a protein that lacks amino acids 313–338 in the transactivation domain [33].

In our laboratory, we screened two populations (healthy and stressed) for their hGR variant profiles and a total of 21 novel splice isoform variations have been identified so far (Fig. 2b). Although the isoforms were illustrated based on the hGRα structure, it should be noted that additional variants may occur due to alternate start sites or exon 9 splicing to produce the hGRβ terminus. The isoforms were given a name with a “hGR” prefix followed by a systematically determined suffix representing the change in the coding DNA sequence. Deletions were indicated by a delta (Δ) followed by the affected exon and, if only a segment of the exon is deleted, the exact number of base pairs missing is indicated. When multiple exons are altered, the numbers are listed in order of 5′ to 3′ and separated with commas. Similarly, insertions were identified using the same naming conventions but with the delta replaced with a hyphen and retained portions of intron were designated using the same format as exonic insertions. The sole departure from this system occurs when the alteration includes an established alternative splice variant (e.g., hGRγ); that segment of the name matched the established variant rather than representing the precise sequence change. Nucleotide changes were also used to describe the variants with the nomenclature proposed by the Human Genome Variation Society (HGVS) (Table 1) [34].

**Table 1:** HGVS nomenclature for novel hGR splice variants

Isoform	RNA [NG_009062.1(NM_000176.2):]	Protein [NG_009062.1(NM_000176.2):]
hGR-H	r.2181_2182ins2181+1_2182-1	p.(Val729Serfs*18)
hGRΔ7(52)	r.1973_2023del	p.(Val658Phefs*6)
hGRΔ3,4	r.1185_1468del	p.(Pro396Serfs*23)
hGR-γ/H	r.[1351_1352ins1351+1_1351+3;2181_2182ins2181+1_2182-1]	p.(Gly451_Gln452insArg;Val729Serfs*18)
hGR-B(59)	r.1184_1185ins1184+9720_1184+9778	p.(Ser395Argfs*2)
hGR-B(122)	r.1184_1185ins1184+15187_1184+15308	p.(Pro396Leufs*9)
hGR-B(1015)	r.1184_1185ins1185-39571_1185-38557	p.(Ser395Argfs*9)
hGR-B(93)	r.1184_1185ins1185-24908_1185-24816	p.(Pro396Thrfs*22)
hGR-B(77)	r.1184_1185ins1185-13046_1185-12970	p.(Pro396Thrfs*22)
hGR-B(54)	r.1184_1185ins1185-9161_1185-9108	p.(Ser395Argfs*9)
hGR-B(93,54)	r.1184_1185ins[1185-24908_1185-24816;1185-9161_1185-9108]	p.(Pro396Thrfs*22)
hGR-B(93,77)	r.1184_1185ins[1185-24908_1185-24816;1185-13046_1185-12970]	p.(Pro396Thrfs*22)
hGR-B(93,46,77)	r.1184_1185ins[1185-24908_1185-24816;1185-20820_1185-20775;1185-13046_1185-12970]	p.(Pro396Thrfs*22)
hGR-B(93,796)	r.1184_1185ins[1185-24908_1185-24816;1185-20552_1185-19757]	p.(Pro396Thrfs*22)
hGR-B(77)/H	r.[1184_1185ins1185-13046_1185-12970;2181_2182ins2181+1_2182-1]	p.(Arg395Serfs*2)
hGR-D(176)	r.1468-1469ins1468+618_1468+793	p.(Ala490Glyfs*18)
hGR-D(177)	r.1468-1469ins1468+649_1468+825	p.(Ala490Aspfs*5)
hGR-D(87)	r.1468-1469ins1468+4219_1468+4305	p.(Ala490Aspfs*16)
hGR-G(44)	r.2023_2024ins2023+5996_2023+6039	p.(Val675Glufs*4)
hGR-G(93)	r.2023_2024ins2024-2043_2024-1951	p.(Val675Glyfs*31)
hGR-G(117)	r.2023_2024ins2024-1586_2024-1470	p.(Val675Glufs*17)

Of our newly reported 21 splice isoforms, several affect large portions, or the entirety, of exons or introns. Our findings include a previously published isoform, hGR-H (previously published as hGR-S1), that retains all 526 bp of intron H, which contains an early stop codon and results in a truncated protein of 745 amino acids [35]. hGRΔ7(52) lacks the 52 nucleotides at the 5′ end of exon 7 and hGRΔ3,4 lacks exons 3 and 4; these code for putative proteins that are 662 and 417 amino acids, respectively. hGR-γ/H has a 3 base pair insertion between exons 3 and 4, matching hGRγ, but also retains intron H, similar to hGR-H.

The remaining 17 novel splice variants are the result of 15 different retained intron sections. For all of them, we verified the presence of splice donor and acceptor sites which confirms their characterization as new cryptic exons. Intron B is the largest intron and predictably yielded the greatest number of variants. This includes hGR-B(59) (59 nucleotides retained), hGR-B(122) (122 nucleotides), hGR-B(1015) (1015 nucleotides), hGR-B(93) (93 nucleotides), hGR-B(77) (77 nucleotides), and hGR-B(54) (54 nucleotides); each new exon contains an early stop codon, resulting in putative proteins of 395, 403, 402, 416, 395, and 402 amino acids, respectively. Combinations of these new exons from intron B were also seen in hGR-B(93,54), which has both the 93 and 54 nucleotide exons, and hGR-B(93,77), which contains both the 93 and 77 nucleotide exons. hGR-B(93,46,77) has the 93 and 77 nucleotide exons flanking a 46 nucleotide exon, and hGR-B(93,796) has the 93 nucleotide exon followed by a 796 nucleotide exon. Additionally, we also found an isoform, hGR-B(77)/H, which has the 77 nucleotide exon and retained intron H. All the splice variants were found in human subjects, except for hGR-B(122) which was found only in tsA201 cells so far.

The other splice variants retain sections from introns D and G. Three new exons were discovered in intron D: one that is 176 nucleotides in length, hGR-D(176); another that is 177 nucleotides, hGR-D(177); and lastly, one that is 87 nucleotides, hGR-D(87). These splice variants code for putative proteins that are 506, 493, and 504 amino acids, respectively, and all are missing portions of the hinge region and the entirety of the LBD. Interestingly, the retained introns from hGR-D(176) and hGR-D(177) have 145 overlapping nucleotides. Lastly, in intron G, hGR-G(44) retains 44 nucleotides, hGR-G(117) retains 117 nucleotides, and hGR-G(93) retains 93 nucleotides, resulting in proteins that are missing a portion of the LBD and are 677, 690, and 704 amino acids, respectively.

### Single nucleotide polymorphisms (SNPs)

**Fig. 3:**
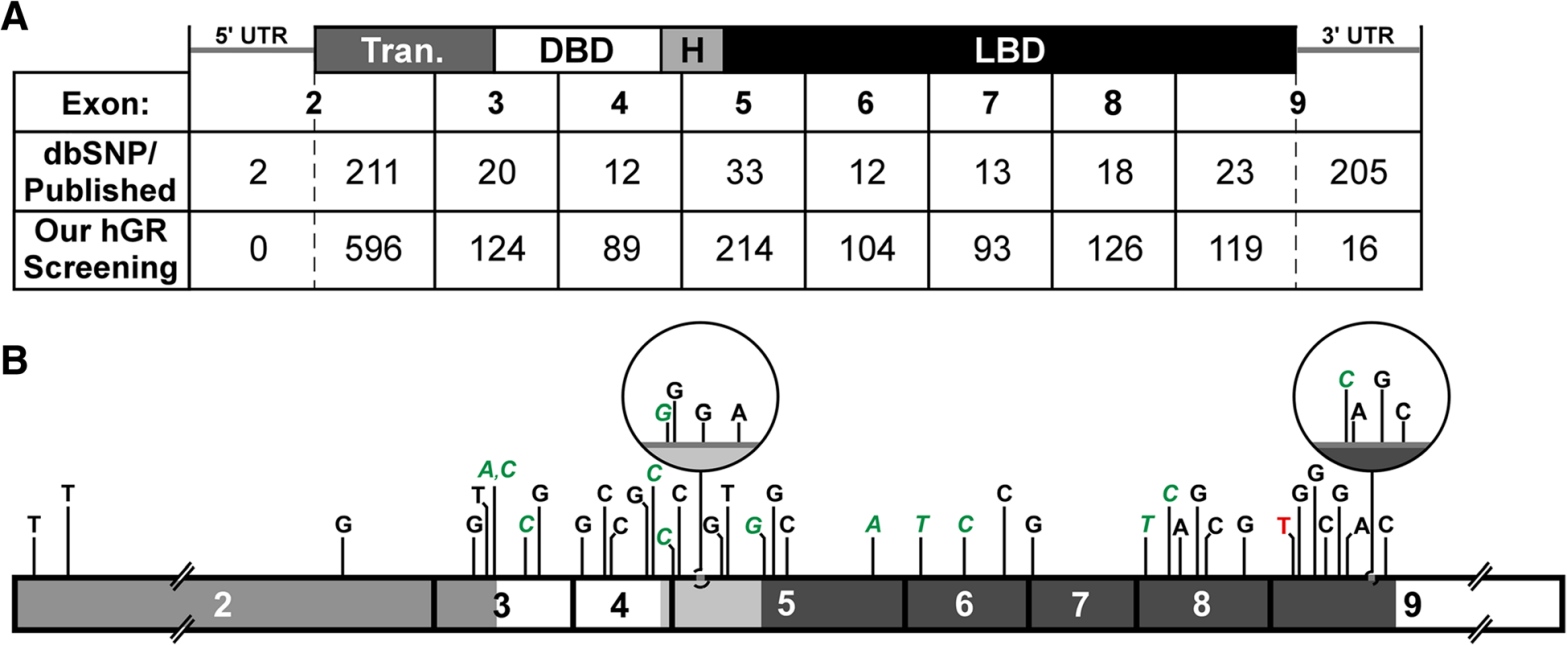
Summary of single nucleotide polymorphisms (SNPs). **b** The table shows the number of SNPs in each exon that were found in our hGR screenings compared to the survey of the dbSNP and publications. Our screenings identified 1481 SNPs in exons 2 through 9: 1465 in the coding regions and an additional 16 in the 3’UTR. The corresponding domains are shown on the top: Transactivation (Tran.), DNA-binding domain (DBD), hinge region (H), and ligand-binding domain (LBD), as well as the 5′ and 3′ untranslated regions (UTR). Intronic SNPs are not shown. **b** A representation of SNPs (found in six or more clones) from our screening is displayed with the nucleotide change indicated above the isoform. Domains are indicated with shading. Green, italic = synonymous substitution; Red = premature termination

**Fig. 4:**
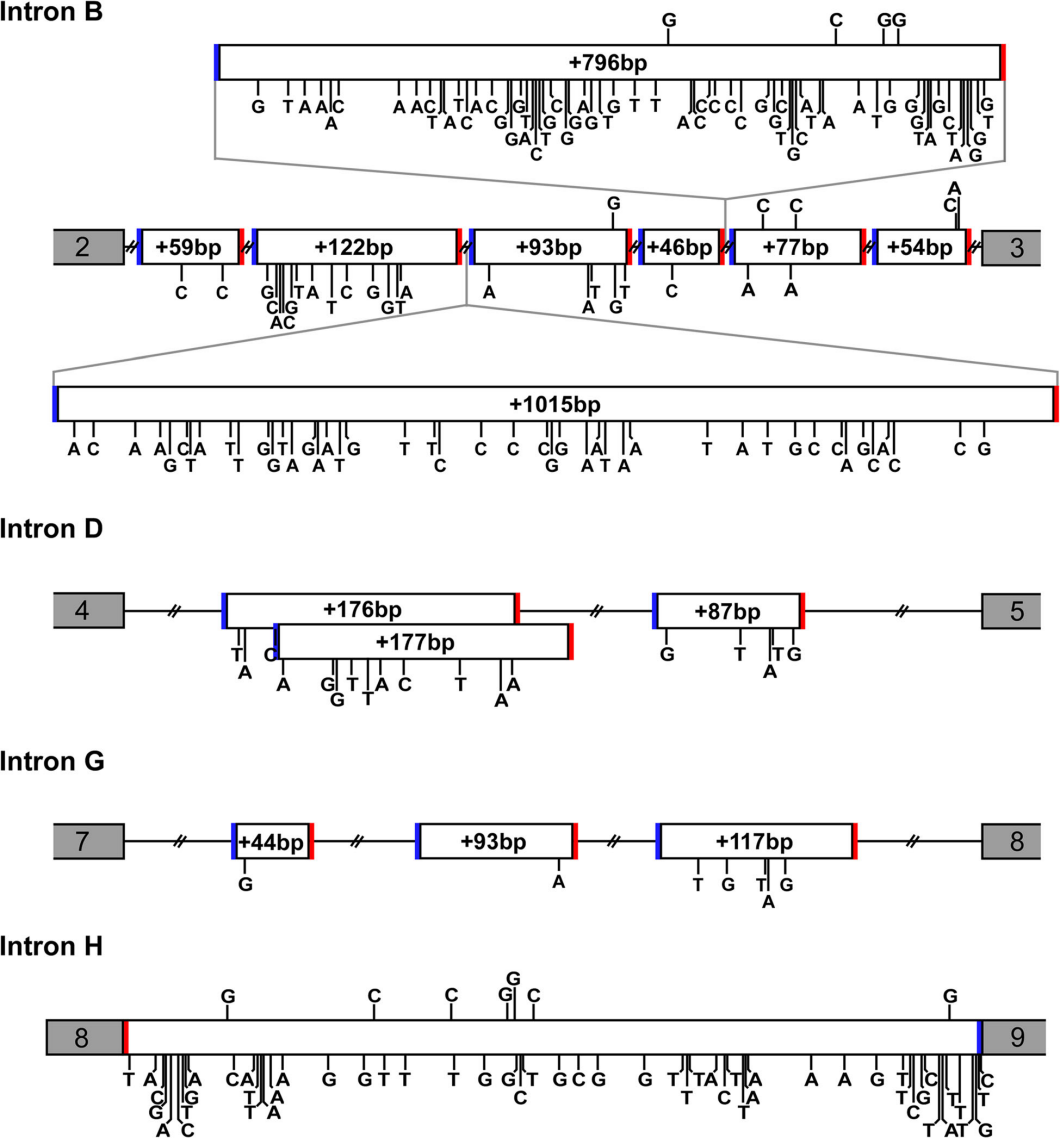
Polymorphisms in new exon sequences. Diagram shows relative positions of retained intron sequences that were found during the hGR screening which includes 14 new exons and intron H. Variations from the reference hGR intron sequence, indicated by a vertical line and substituted nucleotide, are shown above the retained sequence structure if detected in our laboratory’s hGR screening, or below if found in the survey of databases and publications. The two nucleotides immediately before and after the exons are indicated to show the splice acceptor sites (AG; solid blue line) and splice donor sites (GT; solid red line)

From a survey of public databases and publications to look for reported nucleotide substitutions, 758 SNPs have been cataloged (Fig. 3a). The majority of these were cataloged in the National Center for Biotechnology Informatics (NCBI) databases, while an additional three SNPs were reported in other databases and nine SNPs in publications. Out of the 758 SNPs, 342 SNPs are in the coding region, with 226 in the transactivation domain, 16 in the DBD, 16 in the hinge region, and 84 in the LBD. Of those, 226 are non-synonymous, including 5 SNPs which cause premature termination. We also screened portions of the hGR introns to identify SNPs in the regions that correspond to our newly found cryptic exons described above. From this search, 130, 18, and 7 SNPs were found in introns B, D, and G, respectively (Fig. 4). There were 54 documented SNPs in the entirety of intron H (Fig. 4). The remaining SNPs were located in the 5′ and 3′ untranslated regions (UTRs). In the other databases, an additional 28 SNPs were found that had not yet been annotated in NCBI’s SNP database; however, these were located in non-coding areas which are not our focus at this time.

In comparison, our laboratory has identified 1497 SNPs, of which only 124 have been previously reported (Fig. 3a and b). There are 1465 SNPs in the coding region: 647 in the transactivation domain, 153 in the DBD, 101 in the hinge region, and 564 in the LBD. Of those, we found that 478 are synonymous SNPs and 987 are non-synonymous, including 39 that cause premature termination. When screening the hGR intron regions that correspond to our new cryptic exons to detect SNPs, only 9 SNPs were identified in intron B and 7 in intron H (Fig. 4), with the remaining 16 being located in the first 303 base pairs of the 3’UTR. Of the 1497 total SNPs, 718 were found in multiple clones. Due to the small population size, SNP frequency was not evaluated. Additionally, to pursue atypical isoforms, the number of samples sequenced varied among subjects and conditions and thus would not be a fair representation of the frequency of a SNP in a true population.

### Insertions/deletions

**Fig. 5:**
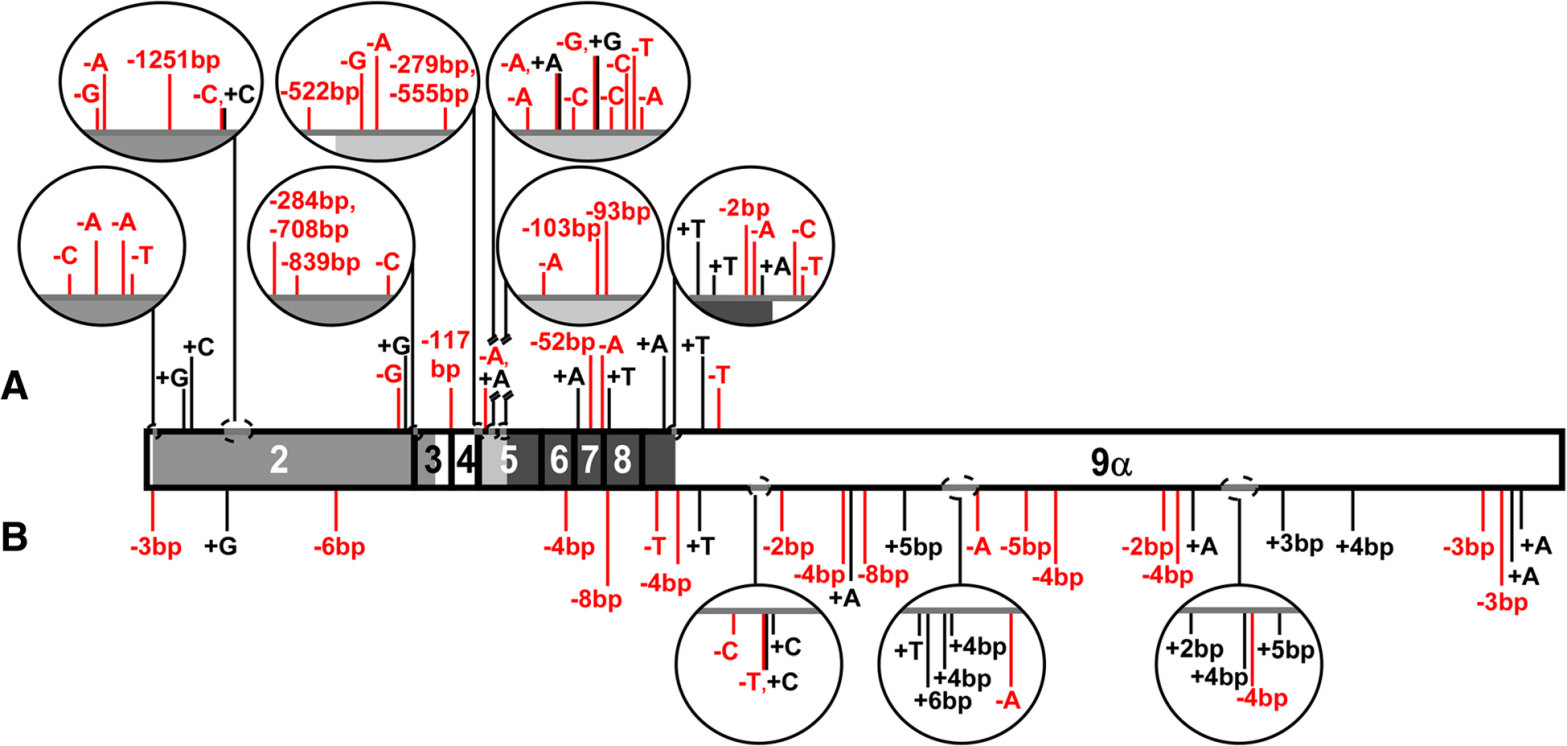
Insertions and deletions in hGR. Additions and deletions are indicated with vertical lines in the hGR sequence. Deletions are shown in red with the nucleotide or number of nucleotides removed, and additions are shown in black with nucleotide or number of nucleotides added. Domains are represented with shading. **a** Additions and deletions found during our laboratory’s screening of hGR (above). **b** Additions and deletions cataloged from the publication and database survey

In addition to SNPs, databases and publications were also screened for insertions and deletions. A total of 25 insertions and 22 deletions were reported (Fig. 5b). Only 3 variants caused frameshifts: 1 single-base-pair insertion in the transactivation domain and 2 multiple-base-pair deletions in the LBD. Two more deletions were in frame, and the remaining insertions and deletions were located in either in introns or the 3’UTR. One deletion/insertion (NM_001018077.1:c.66_68delinsAAA, p.Arg23Lys) was reported solely in the Leiden Open Variation Database (LOVD).

Conversely, our laboratory cataloged 15 insertions and 117 deletions, none of which had been previously reported (Fig. 5a). Of the 15 insertions, 13 were located in exons and therefore caused frameshifts. All 13 were single nucleotide insertions, and only 4 occurred in multiple clones. The additional two single nucleotide inserts were located in non-coding regions, with one insertion in the 3’UTR and the other, which was found in multiple clones, in intron H. Of the 117 deletions, 81 were single nucleotides. The transactivation domain had 27 deletions, the DBD had 9, the hinge region had 47, the LBD had 25, the first 303 base pairs of the 3’UTR had 7, and the remaining 2 deletions occurred in introns. For 38 of the deletions, the variant was found in multiple clones.

### The universal hGR database (UhGR)

**Fig. 6:**
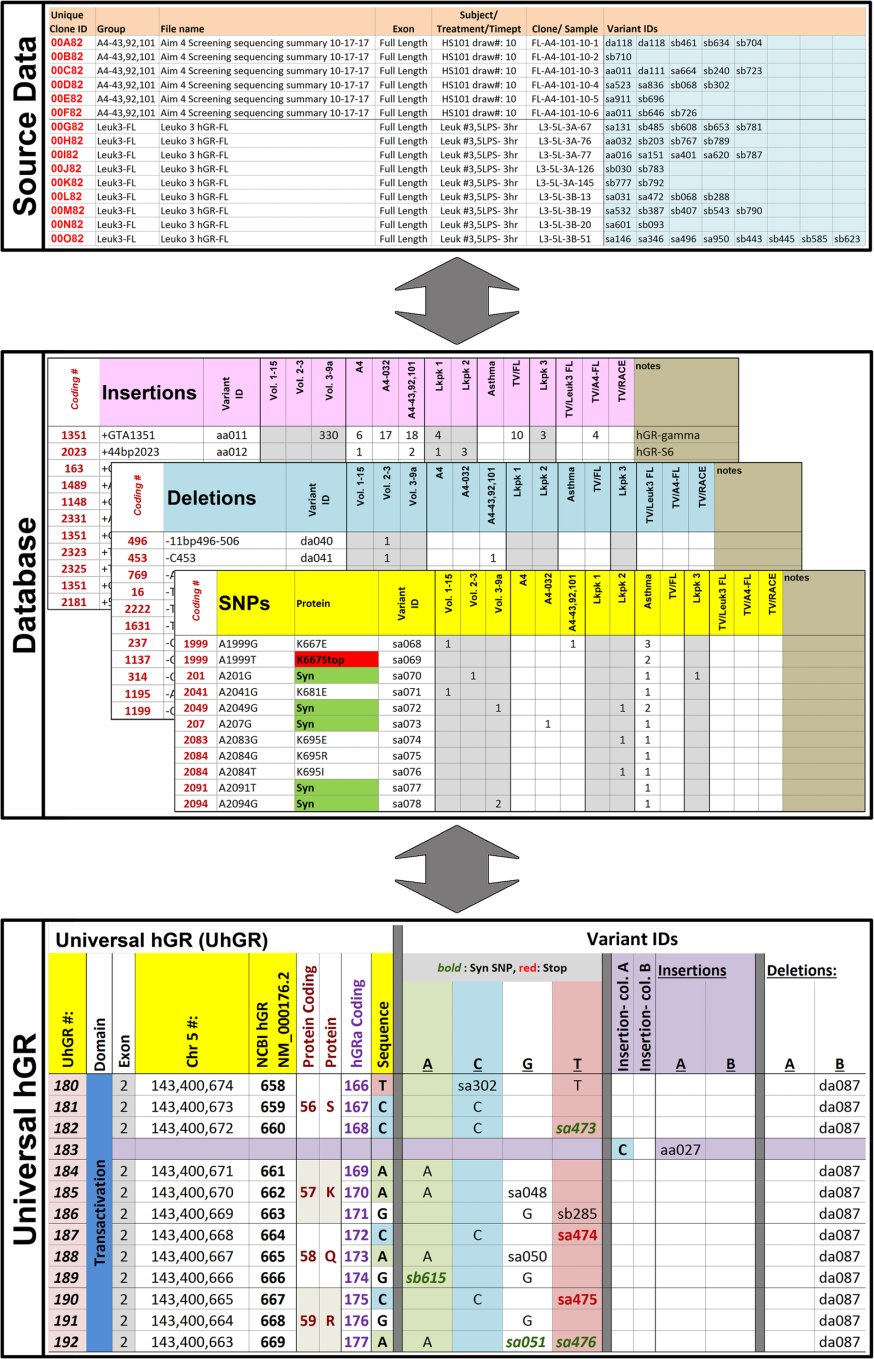
Universal hGR database (screenshots). The different sections comprising the UhGR are shown: list of Source Data; annotated Databases of the insertions, deletions, and SNPs with the unique identifiers corresponding to the clones (CloneID), variants (Variant ID), and dynamically changing hGR location (UhGR #); and the hGR sequence amended with the new variants (Sequence)

To collect and collate the hGR variations identified from the different studies conducted in our laboratory, the Universal hGR (UhGR) database was created. Named the Universal hGR due to the inclusive nature of the database, the UhGR is currently built on a series of Excel worksheets (Microsoft) until a formal software suite can be constructed. The source data consisted of our laboratory hGR clone collection generated from volunteers, burn patients, asthma patients, human Leukopaks, and tsA201 cells. Each clone was assigned a unique identifier (CloneID). Each CloneID was then annotated with information regarding the source (subject demographics, patient disease conditions, PCR amplification conditions, etc.) The clone sequence was then analyzed to identify variants, each of which was assigned a unique identifier (Variant ID). The variant IDs were not unique to each CloneID, so the prevalence of each variant could be determined across the dataset or analyzed for possible clustering within subject groups. Any coding changes (altered amino acid, early termination) were also noted. This information was used to construct the UhGR (Fig. 6).

To visualize the changes in sequence due to the variations, the NCBI reference sequence for hGR (NM_000176.2) was displayed on a spreadsheet within the UhGR. Each variant ID was added to the reference sequence with the nucleotide changes noted; SNPs and deletions were identified in neighboring columns while insertions were directly added to the reference sequence. However, the inclusion of insertions changed the relative positions of each nucleotide within the annotated hGR sequence, so a nucleotide position identifier (UhGR #) column was added to the visualization sheet. Each variant ID in the database was then associated with a UhGR # that dynamically updated whenever more information was added to the hGR sequence. This allows users to easily track the visual changes in the database as more data is accumulated.

The Universal hGR database will be made available to the public and maintained/curated in our laboratory. Although the Universal hGR will ultimately expand to be more than a data management system, many guidelines for LSDBs will be followed [36, 37]. Data will be available to download from a university-hosted website (http://somapp.ucdmc.ucdavis.edu/shriners/uhgr) after users provide basic user information and consent to relevant disclosures, such as indicating that the data is not for diagnostic or commercial purposes. By self-hosting the database, we can ensure its long-term continuance, security, and maintenance on a secure, university server. The database will be regularly backed up to a secondary source to prevent any potential, unforeseeable loss of data. To promote usage, the database will be submitted to LSDB lists, such as https://grenada.lumc.nl/LSDB_list/lsdbs/NR3C1. The database will release updated versions to incorporate new findings from our lab, public databases and publications, and user submitted data. Data from external sources will not be subjected to internal quality control procedures; however, their source information will be provided. The majority of outside variants in the Universal hGR were collected from dbSNP and dbVar and were therefore subjected to NCBI’s own quality control [38, 39].

## Discussion

We are far from understanding hGR regulation and the newly identified variants indicate that the process may be more complex than previously thought. To date, there are over 2000 SNPs, 40 insertions, 139 deletions, and 26 alternative splice variants identified from our screenings and survey of databases and publications. In various combinations, these can form numerous unique hGR isoforms that may be potentially expressed. Although many reported mutations are only linked to subclinical or no manifestations, there are some studies showing deletions [40, 41] and SNPs [42–46] that have been shown to disrupt patient homeostasis; this justifies the need for further investigation into our newly identified isoforms. The expression of one or more of the countless combinations of these variants may explain the highly inconsistent individual responses to stress.

Functional analysis data for several new variants suggests that an individual’s unique hGR profile affects responsiveness to stress and glucocorticoid treatment. An isoform containing one SNP, A829G (NM_000176.2:c.829A > G, p.Lys277Glu), has been shown to result in an in vitro transactivation potential that varied depending on the type and dosage of glucocorticoid treatment [47]. Similarly, isoforms with either the T1463C SNP (NM_000176.2:c.1463 T > C, p.Leu488Pro) or the A2297G SNP (NM_000176.2:c.2297A > G, p.Asn766Ser) were found to have low baseline activity but a hyperactive response, relative to hGRα, when treated with glucocorticoids. Interestingly, while the T1463C isoform was hyperactive when treated with hydrocortisone, methylprednisolone, and dexamethasone, the A2297G isoform showed an augmented response to hydrocortisone treatment only [48]. Although some hGR variants alone are insufficient to elicit a response to glucocorticoids, their presence in combination with other variations can confer a synergistic effect, as demonstrated by the aforementioned A2297G SNP which was originally found in conjunction with A214G (NM_000176.2:c.214A > G, p.Asn72Asp) and T962C (NM_000176.2:c.962 T > C, p.Val321Ala). Single SNP constructs containing either the A214G or the T962C SNPs had little to no baseline activity compared to hGRα; however, when combined with the hyperactive A2297G SNP, the multi-variant isoform had greater activity than any of the single SNP constructs [49]. Although functional characterization is still needed for many more variant isoforms, these initial findings illustrate how these isoforms can alter responsiveness to glucocorticoids in a type-, dose-, and variant combination-dependent manner.

In addition to examining the responsiveness of these new hGR isoforms, the mechanisms of their activity merits further study to understand their impact on differential patient response. The simplest explanation may be that the hGR variants have modified DNA or ligand binding properties [50–53]. The variants could also indirectly affect the function of other transcription factors such as NF-κB and AP-1. The structural changes in the isoforms could also significantly alter receptor degradation, post-translational modification, chaperone binding, or dimerization properties. Another possibility is that the variant receptors utilize an unknown non-canonical activation pathway. Future large studies in mice, which have been shown to also express both GRα and GRβ isoforms [54, 55], may be employed to confirm isoform activity and to shed light on their mechanisms. This will allow us to understand how to influence the expression of these variants in patients and thereby directly impact their personalized treatment regimens.

The vast diversity and multiple functions of the gene is only becoming more apparent as developing technologies and alternative approaches to studying hGR have allowed for more sensitive analysis. Many previous studies have been limited by their focus on cell lines, rather than samples directly from volunteers or patients [56–58]. Additionally, other studies often focus on specific portions of the gene using techniques such as PCR or sequencing, which rely on primers designed to match target sequences and therefore may not detect alternate variants. Many studies also only look at select, previously reported variants [59, 60], or at ones expressed in subjects that present with medical conditions, such as generalized glucocorticoid resistance [61, 62]. By utilizing exon-to-exon screening as well as employing primary cells from human subjects, we were able to detect a significantly greater number of hGR isoform variations in both healthy (volunteers and Leukopaks) and stressed (burn and asthma patients) populations.

Although the frequency of specific variants was not evaluated, a survey of the database reaffirmed the prevalence of several commonly reported variants, such as the N363S SNP (rs56149945), but more importantly it highlights new areas that may be significant in the stress response system. One novel SNP, A1476C (NM_000176.2:c.1476A > C), was frequently found in multiple volunteer samples. The A1476C SNP causes an amino acid change of lysine to asparagine at position 492 [NM_000176.2:p.(Lys492Asn)] in the hinge region. As expected, the two largest domains, the transactivation domain and LBD, have the most variants; however, the hinge region was revealed to have a similar number of deletions despite being significantly smaller. This could indicate that the hinge region, which has been less studied but thought to play a role in protein conformation, may be worth further investigation [63]. Additionally, the abundance of new exons discovered demonstrates that the NR3C1 gene is more complex than the current conventional structure. Similar to hGRβ, the hGR-H variant (with retained intron H) has been detected in all volunteer and patient samples with varying levels of expression [35]. Further research into these areas may help elucidate the role of hGR in individual stress response variations.

As we expand our variant database, it is essential to maintain a dynamic annotation system for the hGR data in order to efficiently analyze the pattern of isoform expression. Due to the large number of variants cataloged, these expression patterns may not be readily apparent. In 2003, when researchers reported on hGR variants aggregated from different sources, the need for a LSDB for the hGR gene, NR3C1, was acknowledged [64]. Although there are a few open source variation databases reported for hGR (https://grenada.lumc.nl/LSDB_list/lsdbs/NR3C1), many of them are defunct. The remaining LSDBs contain data for only a small number of variants, and many have not been kept up-to-date. This leaves only the genome-wide NCBI databases, the SNP database (dbSNP) and the non-SNP variation database (dbVar), which allow for user submission of variants and incorporates data sets from large studies (such as the 1000 Genomes project), as well as the Database of Genomic Variants archive (DGVa) from the European Bioinformatics Institute. The GWAS Central (formerly HGVbase, https://www.gwascentral.org) is also available, but only reports an incomplete list of dbSNP data. Although NCBI’s databases have a sizeable number of variants cataloged, they have several limitations, such as not being intuitive to navigate, accepting non-validated data, and not allowing for demographic information to be submitted to dbSNP or dbVar.

Our UhGR provides a centralized and customized database to store hGR data that will allow for better visualization and analysis of data. Ultimately, the UhGR will become the basis for a formal software suite which will be publically accessible and adaptable for other genes. By supplementing each variant with easy to navigate and sortable information, such as the sample source (e.g. deidentified/anonymized human subject data) and linked changes, trends will be easier to detect. Although the database will not be for diagnostic use, we expect that correlations between variants and clinical data will ultimately reveal patterns which may serve as markers for sepsis and other physiological stresses, as well as help predict individual response to stress and steroid treatment that may prove an important step in personalizing patient therapy regimens.

## Conclusion

Our study indicates that the hGR has more variants at the RNA and DNA levels than previously believed. This information is aggregated in our database, which includes our 21 new splice variants and over 1300 SNPs from primary cells of human subjects. Although more research needs to be done to investigate the mechanisms of how all these hGR variants play a role in divergent responses to a range of stressors, these findings have wide-scale implications in explaining the dynamic and individual-specific variability in responsiveness to glucocorticoid treatment and eventually personalizing patient treatment regimens.

## Methods

### Study populations

All protocols involving the collection, processing, and analysis of human blood samples in this study were approved by the Institutional Review Board of the University of California, Davis. Written informed consent was obtained from participants in this study.

To examine the baseline hGR profiles of healthy volunteer subjects, individuals with a history of major illness (diabetes mellitus, hypertension, chronic obstructive pulmonary disease, inflammatory bowel disease, autoimmune disease, and cancer), pregnant women, and individuals taking exogenous steroids were excluded. The final volunteer population was a mixed cohort of 97 people (70 female and 27 male; ages 20–67) made up of approximately one-half Caucasians, one quarter Asians, with the remaining one quarter being African Americans, Hispanics, or other [49].

To screen for stress-induced hGR variations expressed through a patients’ clinical course, blood was collected from 110 patients with ≥20% total body surface area burns after admission to the UC Davis Medical Center and Shriners Hospitals for Children Burn Units. Samples were collected at regular 2-week intervals as well as additional draws during septic episodes. The 110 patients (82 men and 28 women) were composed of approximately half adults (18 years of age or older). Of the 110 enrolled patients, 18 were selected for further analysis. This group consisted of 13 men and 5 women; and 39% adults and 61% minors. The first eight patients were selected due to their early enrollment in the study, while the remaining patients were selected for having samples with the greatest number of time points.

Additionally, 35 patients with differential responses to steroid treatment were enrolled in an asthma study at the University of California Asthma Network Clinic. As an adjunct study, their genomic DNA was examined for hGR profiles.

Finally, as an in vitro study of stress response, peripheral blood leukocytes were isolated from blood donors using “Leukopaks” purchased from BloodSource (Mather, CA), a local blood bank. Leukopak “1” came from a 50-year-old female, while Leukopaks “2” and “3” were from adult males, ages 53 and 64 respectively. No additional demographic information was provided.

### Identification of hGR isoforms

#### Human subjects (our laboratory)

For the 97 volunteers, the enriched buffy coat samples were erythrocyte-lysed using Buffer EL (Qiagen, Valencia, CA). For the 18 select burn patients, the enriched buffy coat samples were lysed using TRIzol reagent (ThermoFisher, Waltham, MA). Samples from both populations then had RNA extracted using the RNeasy Mini Kit (Qiagen). Reverse transcriptase-polymerase chain reaction (RT-PCR) using the Sensiscript RT Kit (Qiagen) was performed to amplify the hGR coding sequence in two sections (exons 2–3 and exons 3–9) for the volunteers (Additional file 1: Table S1). Using the genomic DNA from asthma patients, each exon was PCR amplified individually. In burn patient samples, RT-PCR was performed using a QuantiTect RT Kit (Qiagen) to amplify full length hGR or sequential hGR exon-to-exon combinations for a more sensitive analysis. RT-PCR was also done using cDNA from tsA201 cells (a transformed HEK293 cell line authenticated by ATCC- via STR profiling,) as a positive protocol control while screening for the cDNA ends. tsA201 cells were a gift from Dr. Daniel Feldman at Shriners Hospitals for Children Northern California. All fragments were cloned into a pGEM-T Easy vector (Promega, Madison, WI) or a pCR-BluntII-TOPO vector (ThermoFisher) then sent for Sanger sequencing at MCLAB (South San Francisco, CA) or GeneWIZ (South Plainfield, NJ). Since Sanger sequencing has distinct advantages relative to alternative methods, such as high accuracy and longer reads, it was determined to be the optimal method to screen a single gene for potentially large variants (e.g., splicing events) in a transcript. Variants were identified by comparison to the National Center for Biotechnology Informatics (NCBI) hGRα reference sequence (NM_000176.2 or NG_009062.1).

#### Leukopaks (our laboratory)

Leukopaks, a white blood cell concentrate prepared from leukoreduced whole blood, were purchased from BloodSource (Sacramento, CA). Peripheral blood mononuclear cells (PBMCs) were separated using a Histopaque-1077 gradient (Sigma, St. Louis MO) and treated with Ammonium-Chloride-Potassium (ACK) buffer to lyse any remaining red blood cells. Cells were resuspended in RPMI-1640 media (ThermoFisher) supplemented with 10% fetal bovine serum (Atlanta Biologicals, Lawrenceville, GA) and seeded in 6-well plates at 9.7 × 10^6^–10.0 × 10^6^ cells per well, then incubated at 37 °C in a 5% CO_2_ atmosphere for 1 h before treatment. Each treatment condition was done in triplicate. To investigate the effect of stress (e.g., exposure to bacterial products as a consequence of burn injury) on hGR isoform expression, cells were exposed to lipopolysaccharide (LPS) from *E. coli* serotype 026:B6 (Sigma). For Leukopak 1, cells were treated with LPS at 5 μg/mL or 10 μg/mL, or with diluent only (i.e. sterile water) as a baseline. For Leukopaks 2 and 3, cells were treated with 5 μg/mL LPS, 1 μM pharmaceutical-grade hydrocortisone sodium succinate (Pfizer, New York, NY), 5 μg/mL LPS with 1 μM hydrocortisone, 10 μg/mL LPS with 1 μM hydrocortisone, or saline diluted with water to match the 5 μg/mL LPS with hydrocortisone condition. Cells were collected after 1, 3, and 13 h. RNA was isolated using the RNeasy Mini Kit (Qiagen), cDNA prepared using the QuantiTect RT Kit (Qiagen), and PCR was performed to amplify both full length hGR and different hGR exon-to-exon combinations (Additional file 1: Table S1). DNA was cloned into a pGEM-T Easy vector (Promega) then sequenced (MCLAB). All variants, relative to the hGRα reference sequence, were documented.

#### Database and publication survey

Several databases were screened for published variants. Using the NCBI Variation Viewer (http://www.ncbi.nlm.nih.gov/variation/), the database of Short Genetic Variations (dbSNP) [13] and the database of Genomic Structural Variation (dbVar) [65] were examined for exons two through nine of the NR3C1 (hGR) gene (NM_000176.2), the intronic sections corresponding to the new cryptic exons, and all of intron H. All reported SNPs, insertions, and deletions in those regions were recorded, along with their corresponding accession numbers (rs#). At this time, duplication events (e.g., copy number variation and short tandem repeat variation) and non-deletion genetic rearrangements (e.g., translocation and inversion) were not included. Other databases screened for those regions were the Global Variome shared LOVD (https://databases.lovd.nl/shared/genes/NR3C1), Leiden Open Variation Database (http://proteomics.bio21.unimelb.edu.au/lovd/genes/NR3C1), and BIPMed SNP Array (http://bipmed.iqm.unicamp.br/snparray/genes/NR3C1).

In addition to databases, a detailed search of PubMed (http://www.ncbi.nlm.nih.gov/pubmed/) was performed for papers published in the past 10 years using the following terms: “human glucocorticoid receptor” AND “polymorphism”, “human glucocorticoid receptor” AND “variant”, “human glucocorticoid receptor” AND “splicing”, “human glucocorticoid receptor” AND “addition”, “human glucocorticoid receptor” AND “deletion”, and “human glucocorticoid receptor” AND “mutation.” Any published variants not in databases were documented.

### hGR database (Universal hGR) construction

**Fig. 7:**
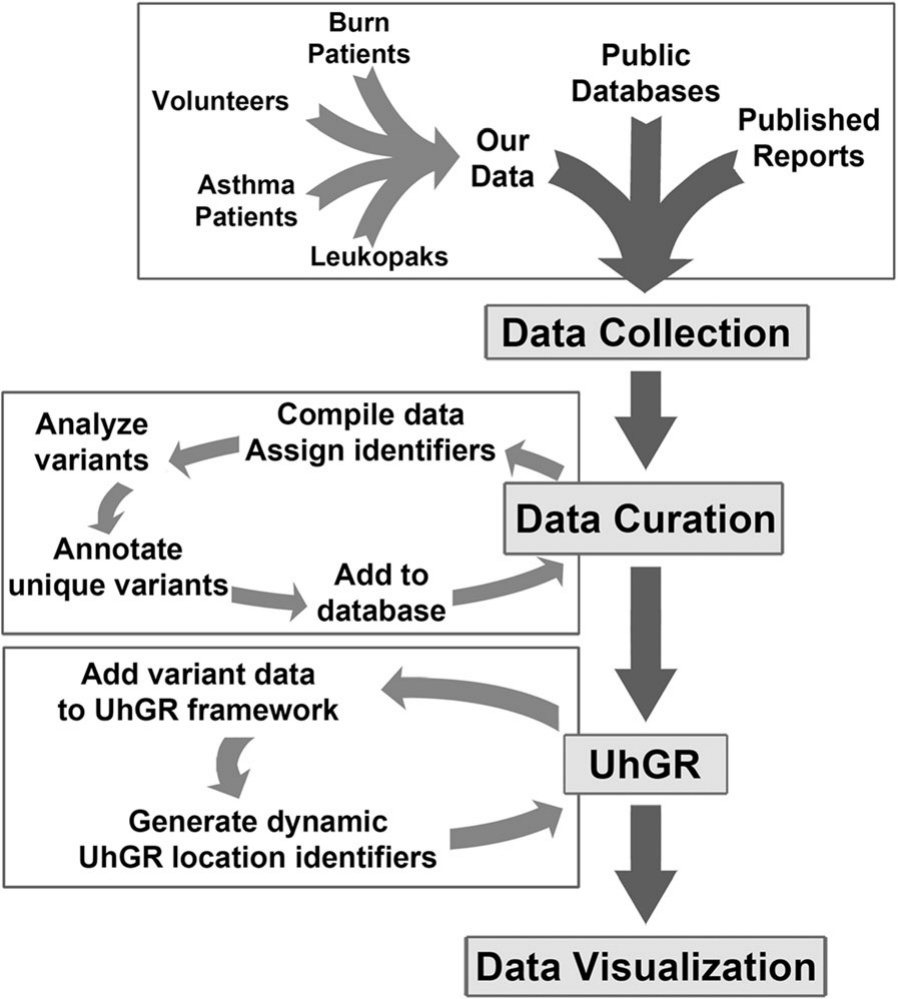
Schema of the Universal hGR database. Data from various sources are collected and curated to identify hGR variants. The analyzed information is assigned unique data tracking identifiers. The changes are appended to the reference hGR sequence, which may result in a revised nucleotide sequence. These changes are annotated with dynamically-adjusting UhGR location identifiers that allow the user to locate specific variants. The adjusted sequence is displayed on an Excel worksheet, along with the lists of variants

For the creation of an hGR-specific database, three data types were used as sources of information for variants: data from published reports, data from available databases, and from our laboratory. This data was organized by initially assigning each data source or clone a unique identifier. Data from each source was mined to identify background information and variants. Each unique change (SNP, insertion, or deletion) was assigned another unique identifier and this information was also added to the database (Fig. 7).

The collected data was used to construct the UhGR framework. Using the NCBI hGR reference sequence (NM_000176.2) as the backbone, basic information such as coding protein and chromosome locations were noted. Variant data (SNP, insertion, and deletion) was annotated onto the sequence. Since the size of the sequence changes after each insertion data is added, a UhGR nucleotide location number was created, which references the current location of the variant. The updated hGR sequence with the annotated variants can then be viewed by the user.

## Additional file

Additional file 1: **Table S1.** PCR primer sequences for hGR screening. (XLSX 13 kb)

## Abbreviations

DBD: DNA-binding domain
dbSNP: Short genetic variations database
dbVar: Database of genomic structural variation
DGVa: Database of genomic variants archive
GR: Glucocorticoid receptor
GRE: Glucocorticoid response element
HBVS: Human Genome Variation Society
hGR: human glucocorticoid receptor
HPA: Hypothalamic-pituitary-adrenal axis
LBD: Ligand-binding domain
LOVD: Leiden open variation database
LSDB: Locus-specific database
NCBI: National Center for Biotechnology Information
PCR: Polymerase chain reaction
rs#: reference SNP cluster ID or accession number
SNP: Single nucleotide polymorphism
UhGR: Universal hGR database
UTR: Untranslated region
